# Omphalomesenteric duct resection using an intraumbilical round incision or a transumbilical vertical incision: report of two cases

**DOI:** 10.1093/jscr/rjaa428

**Published:** 2020-10-28

**Authors:** Yukihiro Tatekawa

**Affiliations:** Department of Pediatric Surgery, Saku Central Hospital Advanced Care Center, Nagano, Japan

**Keywords:** omphalomesenteric duct, Meckel’s diverticulum, intraumbilical, transumbilical

## Abstract

We report our experience with two patients who underwent omphalomesenteric duct resection: one for a patent omphalomesenteric duct and the other for a Meckel diverticulum connected to the umbilicus by a fibrous cord. We used an intraumbilical round incision and a transumbilical vertical incision, respectively. The first patient was a neonate with a patent omphalomesenteric duct who appeared to have a small stoma after ligature of the umbilical cord. Contrast media, injected through a catheter inserted into the stoma, entered the lumen of the small bowel. The second patient was an infant with a Meckel diverticulum connected to the umbilicus by a fibrous cord. After bloody stool was noted, nuclear imaging using 99^m^ technetium pertechnetate revealed a small, round area of intense tracer activity in the midabdomen, suggesting the presence of ectopic gastric mucosa. Using either an intraumbilical or a transumbilical incision is safe and provides good cosmesis.

## INTRODUCTION

The omphalomesenteric duct is a communicating tract between the embryonic yolk sac and the primitive midgut. Persistence of part or all of the omphalomesenteric duct causes a variety of abnormalities related to the intestine and abdominal wall. Persistence of the intestinal end of the omphalomesenteric duct results in Meckel diverticulum [1].

## CASE REPORTS

### Patient 1: A 22-day-old boy

**Figure 1:**
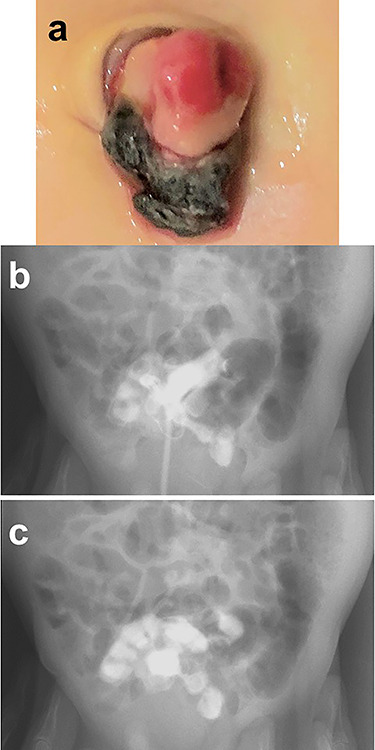
Physical findings on outpatient consultation and trans-stomal fistulography. (**a**) There appears to be a small stoma present after ligation of the umbilical cord. (**b** and **c**) Contrast media, injected through a catheter inserted into the stoma, enters the lumen of the small bowel.

A male infant was born at 39 weeks of gestation, weighing 3036 g. His mother had been treated for Basedow’s disease. After the umbilical cord was ligated, there appeared to be a small stoma present at the base of the cord, with yellowish discharge (Fig. 1a). He was referred to our hospital at 22 days of age. We injected contrast media through a catheter inserted into the stoma and noted that contrast entered the lumen of the small bowel, establishing the diagnosis of a patent omphalomesenteric duct (Fig. 1b and c).

**Figure 2:**
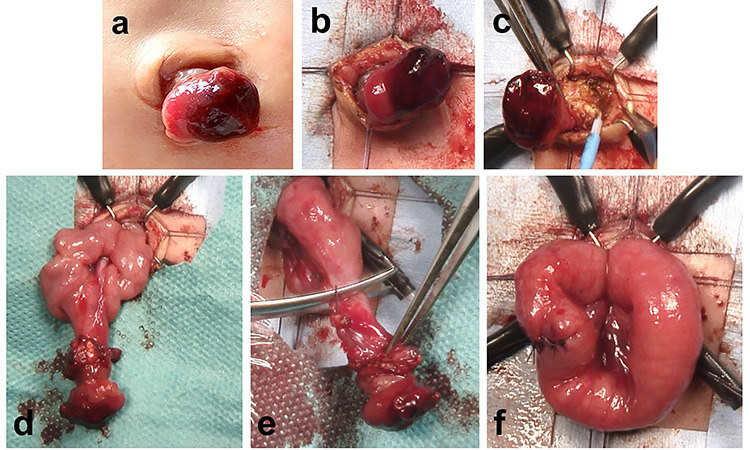
Resection of a patent omphalomesenteric duct using an intraumbilical round incision. (**a**–**c**) The communication between the stoma and the intra-abdominal intestine is cored out from the anterior abdominal wall through an intraumbilical round incision. (**d**) Part of the communication between the patent omphalomesenteric duct and intestine is removed through the intraumbilical incision. (**e** and **f**) The patent omphalomesenteric duct is completely resected, and the cut edge is closed using absorbable sutures.

We used an intraumbilical round incision to resect the patent duct (Fig. 2a–f). The communication between the stoma and the intra-abdominal intestine was cored out from the anterior abdominal wall through the umbilicus (Fig. 2a–c). Part of the communication between the patent omphalomesenteric duct and the intestine was removed through the intraumbilical wound (Fig. 2d). The blood vessel supplying the patent omphalomesenteric duct was divided and ligated. The patent duct was completely resected, and the cut edge was closed using absorbable sutures (Fig. 2e and f). The patient had an uneventful postoperative course and was discharged home. Healing of the umbilical wound resulted in a good cosmetic appearance.

### Patient 2: A 31-month-old girl

**Figure 3:**
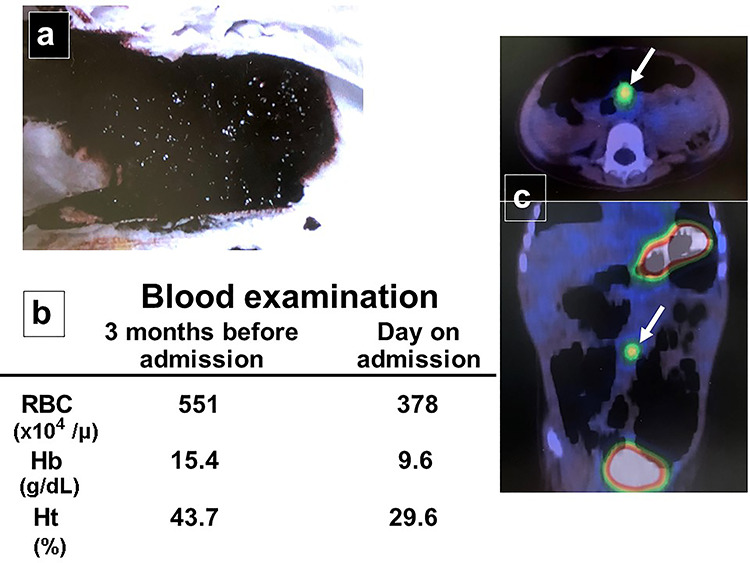
Patient workup. (**a**) Photograph after an episode of bloody stool was noted in the diaper. (**b**) Bloodwork performed 3 months before admission was normal, while laboratory investigation on the day of assessment reveals new-onset anemia. (**c**) Nuclear imaging using 99^m^ technetium pertechnetate reveals a small, round area of intense tracer activity in the midabdomen.

A female infant was born at 37 weeks of gestation, weighing 2068 g. At 31 months of age, her parents noted that she had passed bloody stool twice in the last month (Fig. 3a). She was admitted to the hospital after bloodwork on the day of assessment was compared with routine bloodwork performed 3 months prior and new-onset anemia was noted (Fig. 3b). She underwent nuclear imaging using 99^m^ technetium pertechnetate to investigate the possibility of a Meckel diverticulum. A small, round area of intense tracer activity was noted in the midabdomen, suggesting the presence of ectopic gastric mucosa (Fig. 3c).

**Figure 4:**
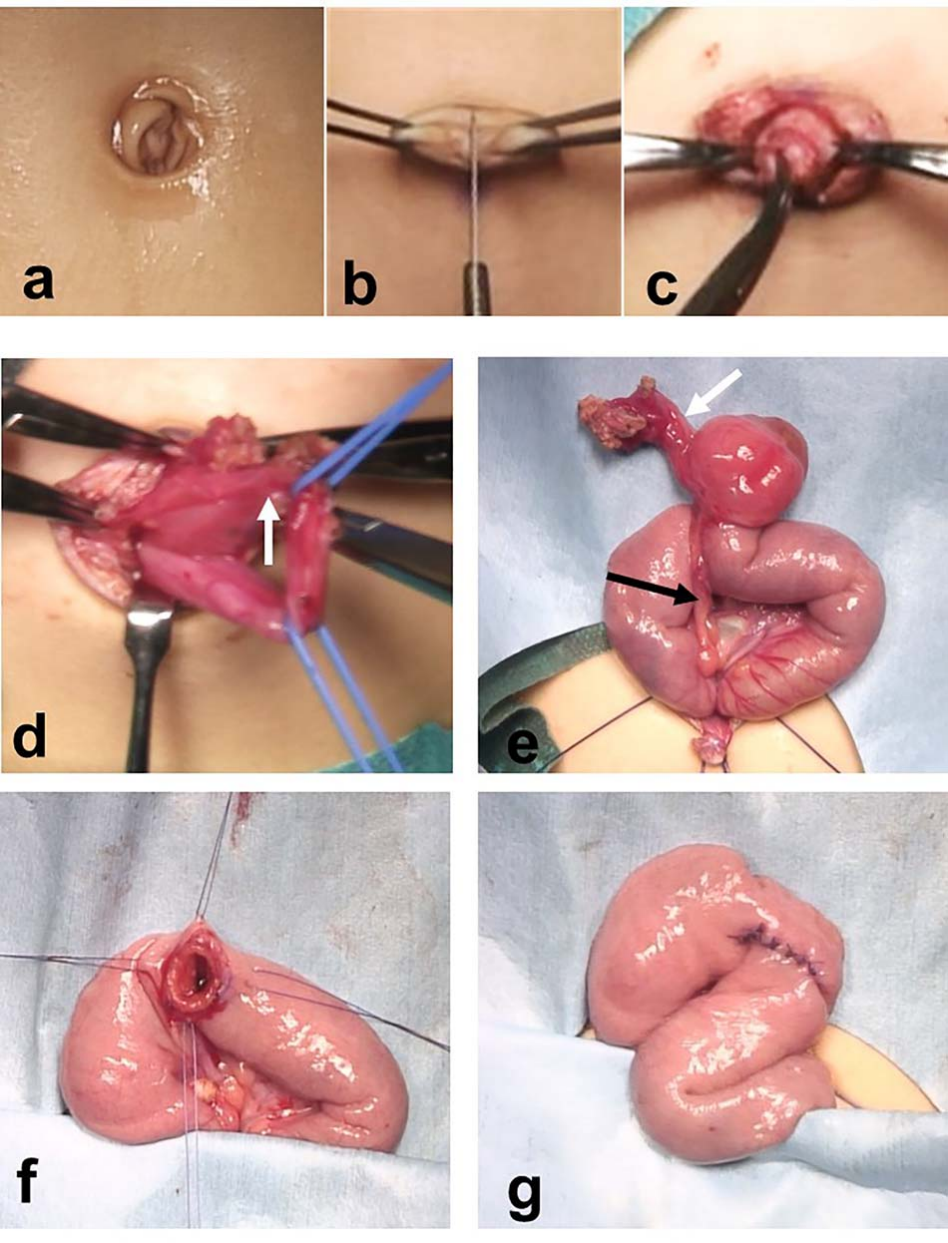
Resection of a Meckel diverticulum connected to the umbilicus by a fibrous cord using a transumbilical vertical incision. (**a-c**) The umbilicus is divided vertically to create a pair of laterally based skin flaps. The subcutaneous tissue is cored out from the anterior abdominal wall through the umbilical incision. (**d**) A fibrous cord connected to a Meckel diverticulum and a urachal remnant (white arrow) is seen. (**e**) The blood vessel supplying the Meckel diverticulum is divided and ligated. (**f** and **g**) Limited segmental resection including the Meckel diverticulum and the fistula is completed. The cut edge is closed using absorbable sutures.

We used a transumbilical vertical incision, dividing the umbilicus vertically to create a pair of laterally based skin flaps, which were then denuded of subcutaneous tissue. More subcutaneous tissue was cored out from the anterior abdominal wall through the umbilical incision (Fig. 4a–c). We then were able to visualize a fibrous cord connected to a Meckel diverticulum with a urachal remnant (Fig. 4d). After ligation and resection of the urachal remnant, we divided and ligated the blood vessels supplying the Meckel diverticulum (Fig. 4e). We performed a limited segmental resection of the Meckel diverticulum and fistula, and closed the cut edge using absorbable sutures (Fig. 4f and g). Subsequent histologic examination of the specimen showed ectopic gastric mucosa. The patient had an uneventful postoperative course and was discharged home. Healing of the umbilical wound resulted in a good cosmetic appearance.

## DISCUSSION

Abnormalities resulting from persistence of the omphalomesenteric duct are classified into four types: Meckel diverticulum, omphalomesenteric duct cyst, patent omphalomesenteric duct and Meckel diverticulum connected to the umbilicus by a fibrous cord [1]. Our patients show the two types: persistent omphalomesenteric duct with an enterocutaneous fistula, and fibrous cord between the small intestine and the posterior surface of the umbilicus and Meckel diverticulum [2].

The diagnosis of a patent omphalomesenteric duct can be established by fistulography through the stoma. We found that an intraumbilical round incision is useful and allows for effective coring out and removal of the fistula from the anterior abdominal wall. It is easy to excise the patent omphalomesenteric duct, and the postoperative wound has a good cosmetic appearance [3]. Other surgical approaches used for this condition include the semicircular infraumbilical incision, supraumbilical transverse incision and infraumbilical transverse incision [3, 4]. However, methods other than the intraumbilical round incision may lead to scarring outside the umbilicus and create a more invasive operative wound.

Laparoscopic surgery is recommended when nuclear imaging confirms the possibility of a Meckel diverticulum. We use transumbilical vertical incisions for laparoscopic appendectomies and laparoscopic excision of urachal sinuses in pediatric patients [5]. In our patient with a symptomatic Meckel diverticulum, we planned to perform laparoscopic surgery and were therefore able to recognize the remnant of the urachus and the omphalomesenteric duct. It is important to take care not to damage the umbilical remnant when inserting the laparoscopic instruments on performing laparoscopic surgery.

Meckel diverticulum contains ectopic gastric mucosa and, when associated with a symptomatic omphalomesenteric duct, causes rectal bleeding. Nuclear imaging using 99^m^ technetium pertechnetate is useful for diagnosis, with a specificity and sensitivity of 85 and 95% in children; in adults, the sensitivity and specificity are around 60 and 9% [6]. False-negative results are attributed to the diverticulum containing mucosa other than gastric, necrosis of the mucosa and rapid peristalsis. Various pharmacologic agents such as oral cimetidine or intravenous glucagon are used to enhance the sensitivity of Meckel scintigraphy.

Other symptomatic presentations of omphalomesenteric ducts include intestinal obstruction with a vitelline ligament of a Meckel diverticulum or an omphalomesenteric cyst. Intestinal occlusion may result from adhesions, volvulus, intussusception or internal hernia from a patent omphalomesenteric duct or from a fibrous connection between the umbilicus and the intestine [4, 7–9].
